# Conjugate Heat Transfer in Rayleigh-Bénard Convection in a Square Enclosure

**DOI:** 10.1155/2014/786102

**Published:** 2014-05-28

**Authors:** Habibis Saleh, Ishak Hashim

**Affiliations:** ^1^School of Mathematical Sciences, Faculty of Science & Technology, Universiti Kebangsaan Malaysia (UKM), 43600 Bangi, Selangor, Malaysia; ^2^Solar Energy Research Institute, Universiti Kebangsaan Malaysia (UKM), 43600 Bangi, Selangor, Malaysia; ^3^Department of Mathematics, Faculty of Science, King Abdulaziz University, P.O. Box 80257, Jeddah 21589, Saudi Arabia

## Abstract

Conjugate natural convection-conduction heat transfer in a square enclosure with a finite wall thickness is studied numerically in the present paper. The governing parameters considered are the Rayleigh number (5 × 10^3^ ≤ Ra ≤ 10^6^), the wall-to-fluid thermal conductivity ratio (0.5 ≤ Kr ≤ 10), and the ratio of wall thickness to its height (0.2 ≤ *D* ≤ 0.4). The staggered grid arrangement together with MAC method was employed to solve the governing equations. It is found that the fluid flow and the heat transfer can be controlled by the thickness of the bottom wall, the thermal conductivity ratio, and the Rayleigh number.

## 1. Introduction

The beautiful hexagonal patterns can be obtained by a simple experiment first conducted by Henri Bénard, a French physicist, in 1900. Later, in 1916, Lord Rayleigh proposed his theory of a feedback coupling resting on buoyancy: a fluid particle hotter than its environment encounters ever colder fluid as it rises, which leads to the instability. The critical Rayleigh numbers, which signal the onset of natural convection in rectangular boxes heated from below and cooled from above, have been obtained theoretically by Davis [1] and Catton [2]. Corcione [3] proposed the heat transfer correlations in terms of the average Nusselt number with Rayleigh number and the aspect ratio of the rectangular enclosure heated from below. This convection problem has attracted a great deal of attention from researchers because of its presence in both nature and industrial applications. In nature, the convection cells formed from air rising above sunlight-warmed land or water are a major feature of all weather systems. Convection is also seen in the rising plume of hot air from fire, oceanic currents, and sea-wind formation. A very common industrial application of natural convection is free air cooling without the aid of fans: this can happen on small scales (computer chips) to large scale process equipment.

The literatures indicated that most of the reported works on Rayleigh-Bénard convection do not study the effect of a conductive bottom wall. Applications of conductive bottom wall can be found, for example, in high performance insulation for buildings. When the conductivities of the wall and fluid are comparable and the wall thickness is finite, conduction-convection analysis is necessary. This coupled conduction-convection problem is known as conjugate convection. Conjugate natural convection in a rectangular enclosure surrounded by walls was firstly examined by Kim and Viskanta [4, 5]. Their results show that wall conduction effects reduce the average temperature differences across the cavity, partially stabilize the flow, and decrease the heat transfer rate. A differentially heated vertical square enclosure with two finite thickness horizontal walls was investigated by Mobedi [6]. Kaminski and Prakash [7] and Misra and Sarkar [8] performed a numerical study on conjugate convection in a square cavity with a thick conducting wall on one of its vertical sides. The influence of wall conduction on natural convection in an inclined square cavity was researched by Acharya and Tsang [9]. The effects of the Rayleigh number, dimensionless conductivity ratio, dimensionless wall width, and inclination angle on the natural convection in an inclined enclosure bounded by a solid wall were investigated by Ben Yedder and Bilgen [10]. Later, Nouanegue et al. [11] extended the work of Ben Yedder and Bilgen [10] to include a radiation effect. Zhang et al. [12] studied conjugate convection in an enclosure with time-periodic sidewall temperature and inclination. Recently, Aminossadati and Ghasemi [13] investigated conjugate convection in an inclined nanofluid-filled enclosure.

The investigation of the effect of the conductive bottom wall on convective flows in a square enclosure has not received much attention. Recent works include those of Varol et al. [14] and Saleh et al. [15] for Darcy-Bénard convection in a porous enclosure. The aim of this work is to examine the effect of the conductive bottom wall on Rayleigh-Bénard convection in a square enclosure. This effect on the flow development, temperature distribution, and heat transfer rate in the wall and fluid will be presented graphically.

## 2. Mathematical Formulation

**Figure 1:**
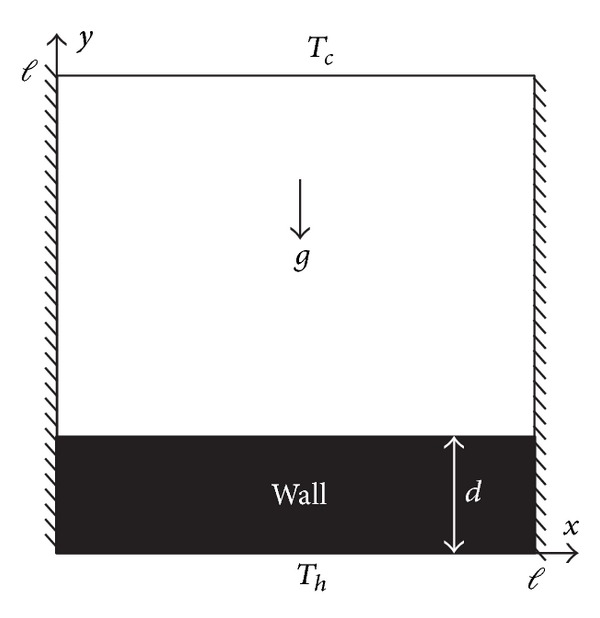
Schematic representation of the model.

A schematic diagram of a square enclosure with a finite wall thickness is shown in Figure 1. The bottom surface of the impermeable wall is heated to a constant temperature *T*_*h*_, and the top surface of the enclosure is cooled to a constant temperature *T*_*c*_, while the vertical walls are kept adiabatic.

Thermophysical properties of the fluid in the flow field are assumed to be constant except the density variations causing a body force term in the momentum equation. The Boussinesq approximation is invoked for the fluid properties to relate density changes to temperature changes and to couple in this way the temperature field to the flow field. Under the above assumptions, the governing equations for steady natural convection flow using conservation of mass, momentum, and energy can be written as

(1) $$\begin{array}{c} \frac{\partial u}{\partial x}+\frac{\partial v}{\partial y}=0,\\ u\frac{\partial u}{\partial x}+v\frac{\partial u}{\partial y}=-\frac{1}{\rho }\frac{\partial p}{\partial x}+\nu \left(\frac{\partial^{2}u}{\partial x^{2}}+\frac{\partial^{2}u}{\partial y^{2}}\right),\\ u\frac{\partial v}{\partial x}+v\frac{\partial v}{\partial y}=-\frac{1}{\rho }\frac{\partial p}{\partial x}+\nu \left(\frac{\partial^{2}v}{\partial x^{2}}+\frac{\partial^{2}v}{\partial y^{2}}\right)+g\beta \left(T_{f}-T_{c}\right),\\ u\frac{\partial T_{f}}{\partial x}+v\frac{\partial T_{f}}{\partial y}=\alpha \left(\frac{\partial^{2}T_{f}}{\partial x^{2}}+\frac{\partial^{2}T_{f}}{\partial y^{2}}\right) \end{array}$$

and the energy equation for the impermeable wall is

(2) $$\begin{array}{c} \frac{\partial^{2}T_{w}}{\partial x^{2}}+\frac{\partial^{2}T_{w}}{\partial y^{2}}=0, \end{array}$$

where the subscripts *f* and *w* stand for the fluid and the wall, respectively. No-slip condition is assumed at all of the solid-fluid interfaces. Using the following nondimensional variables:

(3) $$\begin{array}{c} X=\frac{x}{l},\;\;Y=\frac{y}{l},\;\;U=\frac{ul}{\alpha },\\ V=\frac{vl}{\alpha },\;\;\Theta_{f}=\frac{T_{f}-T_{c}}{T_{h}-T_{c}},\;\;\Theta_{w}=\frac{T_{w}-T_{c}}{T_{h}-T_{c}},\\ P=\frac{pl^{2}}{\rho \alpha^{2}},\;\;\text{Pr}=\frac{\nu }{\alpha },\;\;\text{Ra}=\frac{g\beta \left(T_{h}-T_{c}\right)l^{3}\text{Pr}}{\nu^{2}}, \end{array}$$

the resulting nondimensional forms of (1)-(2) are the following:

(4) $$\begin{array}{c} \frac{\partial U}{\partial X}+\frac{\partial V}{\partial Y}=0,\\ U\frac{\partial U}{\partial X}+V\frac{\partial U}{\partial Y}=-\frac{\partial P}{\partial X}+\text{Pr}\left(\frac{\partial^{2}U}{\partial X^{2}}+\frac{\partial^{2}U}{\partial Y^{2}}\right),\\ U\frac{\partial V}{\partial X}+V\frac{\partial V}{\partial Y}=-\frac{\partial P}{\partial X}+\text{Pr}\left(\frac{\partial^{2}V}{\partial X^{2}}+\frac{\partial^{2}V}{\partial Y^{2}}\right)+\text{RaPr}\Theta_{f},\\ U\frac{\partial \Theta_{f}}{\partial X}+V\frac{\partial \Theta_{f}}{\partial Y}=\left(\frac{\partial^{2}\Theta_{f}}{\partial X^{2}}+\frac{\partial^{2}\Theta_{f}}{\partial Y^{2}}\right),\\ \frac{\partial^{2}\Theta_{w}}{\partial X^{2}}+\frac{\partial^{2}\Theta_{w}}{\partial Y^{2}}=0. \end{array}$$

The values of the nondimensional velocity are zero in the wall region and on the solid-fluid interfaces. The boundary conditions for the nondimensional temperatures are the following:

(5) $$\begin{array}{c} \Theta_{w}\left(X,0\right)=1;\;\;\Theta_{f}\left(X,1\right)=0,\\ \frac{\partial \Theta_{f}\left(0,Y\right)}{\partial X}=0;\;\;\frac{\partial \Theta_{w}\left(0,Y\right)}{\partial X}=0,\\ \frac{\partial \Theta_{f}\left(1,Y\right)}{\partial X}=0;\;\;\frac{\partial \Theta_{w}\left(1,Y\right)}{\partial X}=0,\\ \Theta_{f}\left(X,D\right)=\Theta_{w}\left(X,D\right);\;\;\frac{\partial \Theta_{f}\left(X,D\right)}{\partial Y}=\frac{\text{Kr}\partial \Theta_{w}\left(X,D\right)}{\partial Y}, \end{array}$$

where Kr = *k*_*w*_/*k*_*f*_ is the thermal conductivity ratio. The physical quantities of interest in this problem are the average Nusselt number defined by

(6) $$\begin{array}{c} {\bar{\text{Nu}}}_{w}=\overset{1}{\underset{0}{\int }}{\frac{-\partial \Theta_{w}}{\partial Y}|}_{Y=0,D}\text{d}Y,\\ {\bar{\text{Nu}}}_{f}=\overset{1}{\underset{0}{\int }}{\frac{-\partial \Theta_{p}}{\partial Y}|}_{Y=D,1}\text{d}Y, \end{array}$$

where ${\bar{\text{Nu}}}_{w}$ represents the dimensionless heat transfer through the walls.

## 3. Computational Methodology

Staggered grid arrangement together with the Marker and Cell (MAC) method [16] is adopted to solve the governing equations (4) subject to the boundary conditions (5). Due to the lack of boundary conditions for pressure, the use of the staggered grid and MAC formulation provides an advantage. That is, one may locate the secondary grid along the boundaries of the domain where only specification of velocity boundary conditions is required but not of the pressure. The fictitious values of velocity outside the domain are obtained by extrapolation of the interior points as given by Hoffmann and Chiang [17]. The temperature conditions at the interface boundary are

(7) (Θw)i,ND+1 =[(1Kr)(−(Θf)i,ND+3+4(Θf)i,ND+2−3(Θf)i,ND+1)+4(Θw)i,ND−(Θw)i,ND]×(3)−1,

where the subscript *i* is used to represent the coordinate *X* and ND is the number of nodal points in the *Y*-axis in the wall.

**Figure 2:**
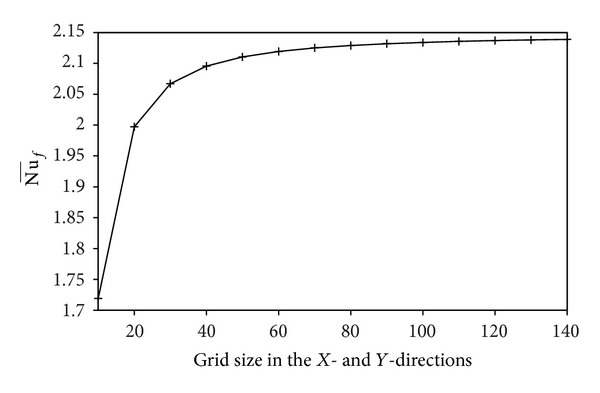
Grid independency study: ${\bar{\text{Nu}}}_{f}$ versus number of grid points at *D* = 0.1, Kr = 0.5, and Ra = 10^5^.

Regular and uniform grid distribution is used for the whole enclosure. The effect of grid resolution was examined in order to select the appropriate grid density as demonstrated in Figure 2. The results indicate that 120 × 120 grid can be used in the final computations. To validate the computational code, the previously published problems on conjugate natural convection in a square cavity with a conducting side wall were solved.  Table 1 shows the comparison of the average Nusselt number between the present results and the available results found in the literature for various Rayleigh numbers and thermal conductivity ratios. The comparison was in excellent agreement with the results reported by [7, 18, 19]. These comprehensive verification efforts demonstrated the robustness and accuracy of the present computation.

## 4. Results and Discussion

The analyses in the undergoing numerical investigation are performed in the following range of the associated dimensionless groups: the wall thickness, 0.02 ≤ *D* ≤ 0.4, the thermal conductivity ratio, 0.5 ≤ Kr ≤ 10, and the Rayleigh number, 5 × 10^3^ ≤ Ra ≤ 10^6^.

**Figure 3:**
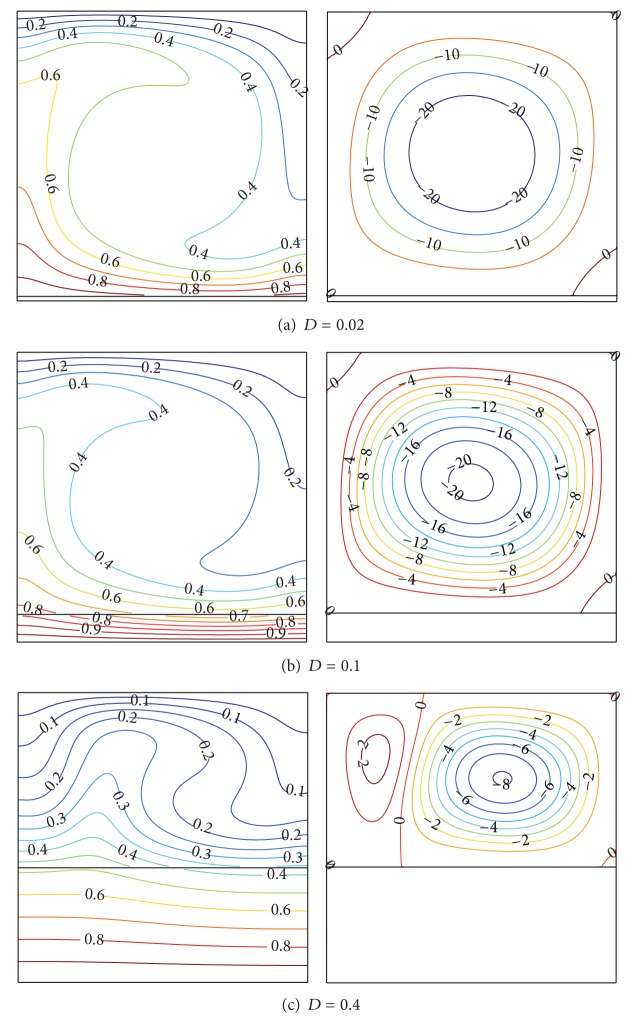
Isotherms (left) and streamlines (right) at Ra = 10^5^ and Kr = 1.

Figure 3 illustrates the effects of the wall thickness parameter *D* for Ra = 10^5^ and Kr = 1 on the thermal fields and flow fields in the fluid and in the bottom solid wall. As can be seen, the parameter *D* affects the fluid and the solid temperatures as well as the flow characteristics. The strength of the flow circulation of the fluid is much higher for a thin solid bottom wall. A circular main cell is formed at a thin bottom; then, the main cell shape becomes elliptical and finally breaks up into dual contrarotative cells at *D* = 0.4. This is because the fluid adjacent to the hotter wall has lower density than the fluid at the middle plane. As a result, the fluid moves upward due to the Archimedes force from the middle portion of the top wall. When the fluid reaches the upper part of the enclosure, it is cooled, so its density increases; then, the fluid flows downward at the left and the right planes of the enclosure. This creates a successive cell that is well known as Bénard cells. It is important to note that the Rayleigh number in the present study is based on the total height of the enclosure.

**Figure 4:**
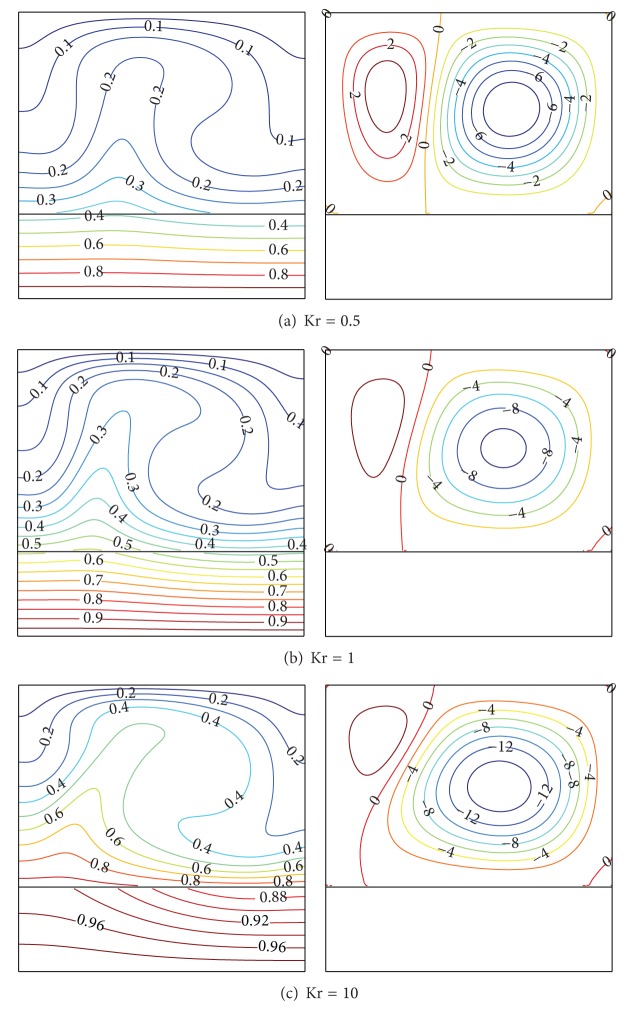
Isotherms (left) and streamlines (right) at Ra = 10^5^ and *D* = 0.3.

To show the effect of the thermal conductivity ratio Kr on the thermal fields and the circulation of the fluid in the enclosure, the isotherms and streamlines are presented in Figure 4 for Ra = 10^5^ and *D* = 0.3. Three different conductivity ratios are selected: Kr = 0.5, Kr = 1, and Kr = 10. It is observed that two contrarotative cells are formed as shown in Figures 4(a)–4(c). The clockwise (“−” sign) circulation cell refers to natural circulation; that is, the main cell and the counterclockwise (“+” sign) circulation cell refer to the secondary cell. As the conductivity ratio increases, the magnitude of the main cell increases, while the magnitude of the secondary cells decreases and shrinks. This phenomenon is due to the temperature gradient near the wall that increases with the increase of the parameter Kr. Thus, much heat transfer from the bottom solid wall to the fluid is obtained for higher values of Kr (good conductive solid wall). It is also observed that convection effects in the fluid become stronger for higher values of Kr.

**Figure 5:**
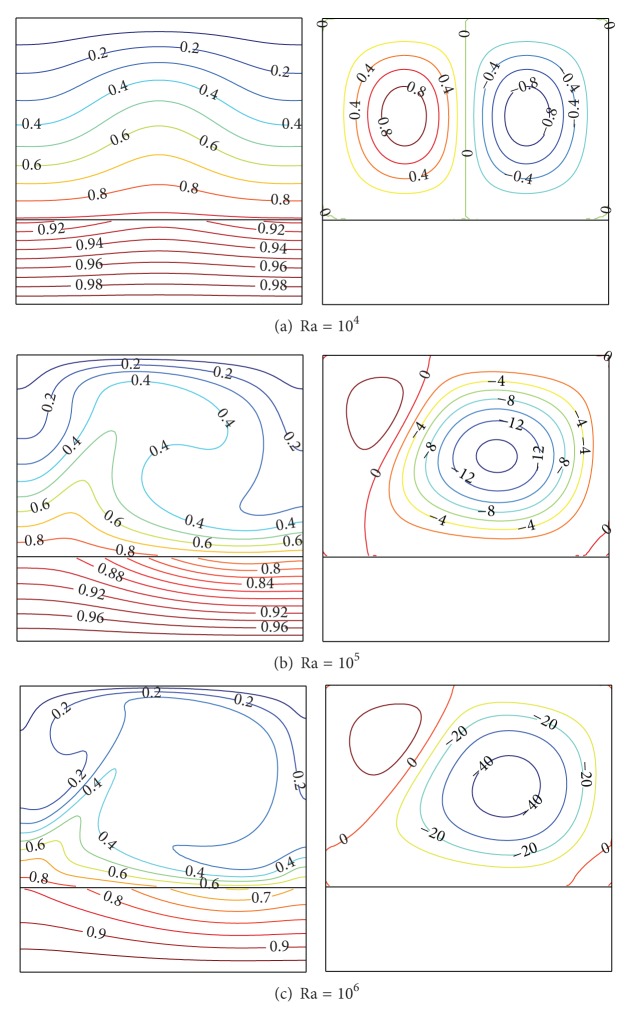
Isotherms (left) and streamlines (right) at *D* = 0.3 and Kr = 5.

Figures 5(a)–5(c) show the effects of Ra on the thermal fields and flow fields in the porous enclosure and in the bottom solid wall with constant values of *D* = 0.3 and Kr = 5. As can be seen in Figure 5(a), two contrarotative cells were formed with the same size and strength. When Ra takes higher values as depicted in Figures 5(b) and 5(c), the main cell circulation strengthens while the secondary (left) one weakens and shrinks. Thermal fields show that the temperature distribution is almost uniform in the solid bottom wall for all values of Ra investigated. The thermal fields in the fluid are modified strongly by increasing Ra as shown in Figures 5(b) and 5(c). This refers to the strength of convection current related to the Ra values.

**Figure 6:**
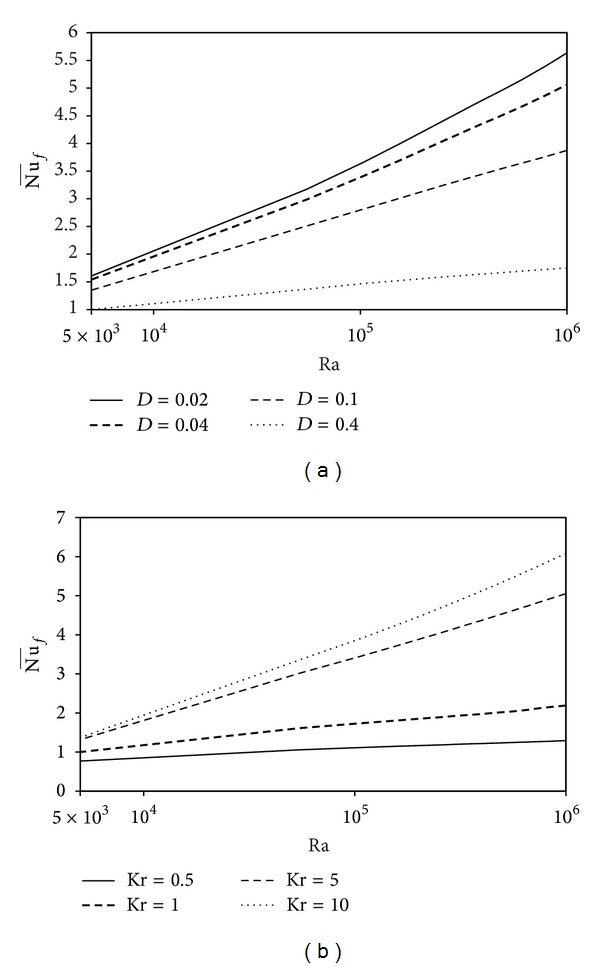
Variations of ${\bar{\text{Nu}}}_{f}$ with Ra for different (a) *D* and (b) Kr.

**Figure 7:**
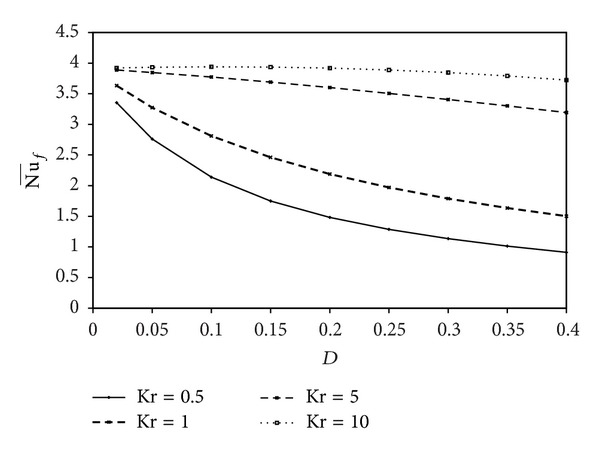
Variations of ${\bar{\text{Nu}}}_{f}$ with *D* for different Kr.

Variations of the average Nusselt number with the Rayleigh number are shown in Figure 6(a) for different values of the wall thickness *D* and thermal conductivity ratio, Kr = 1. The result presented in Figure 6(a) shows that, for a thin solid wall, the heat transfer from the fluid increases with increasing Ra. This is due to the increasing of domination of convection heat transfer by increasing the buoyancy force inside the fluid. Figure 6(a) also shows that ${\bar{\text{Nu}}}_{f}$ becomes constant for the highest values of the thickness parameter of the solid bottom wall. Variations of the average Nusselt number with the Rayleigh number are shown in Figure 6(b) for different values of Kr with constant *D* = 0.3. Obviously, the heat transfer increases by increasing Ra. The heat transfer enhancement by increasing Ra is more pronounced at higher values of thermal conductivity ratio as shown in Figure 6(b). This is due to the temperature gradient near the solid wall that increases with increasing Kr as shown in Figure 4. Variations of the average Nusselt number with the wall thickness are presented in Figure 7 for different values of Kr with constant Ra = 10^5^. This figure shows that the heat transfer decreases by increasing the solid wall thickness *D*. There is a considerable difference between the heat transfer for small and large values of Kr. For Kr = 0.5, the heat transfer is almost constant. When Kr takes higher values, heat transfer drops sharply by increasing *D* and becomes a conduction mode. This is due to the bottom solid wall that behaves as an insulated material in this case. Finally, the average Nusselt number can be correlated pretty well with the wall thickness, the thermal conductivity ratio, and the Rayleigh number as follows:

(8) $$\begin{array}{c} \bar{\text{Nu}}=0.1269D^{-0.2155}{\text{Kr}}^{0.3228}{\text{Ra}}^{0.2118}. \end{array}$$

This correlation is valid for 5 × 10^4^ ≤ Ra ≤ 10^6^, 0 ≤ *D* ≤ 0.4, and 0.5 ≤ Kr ≤ 10. Note that the maximum error is less than 5% for the valid ranges.

## 5. Conclusions

The present numerical simulations study the effects of conduction in bottom wall on Rayleigh-Bénard convection in a square enclosure. The dimensionless forms of the governing equations were solved using the finite difference method. Detailed computational results for flow and temperature fields and the heat transfer rates in the enclosure have been presented in graphical forms. The main conclusions of the present analysis are as follows.

1. The strength of the flow circulation of the fluid is much higher with thin walls and/or higher value of the solid to fluid thermal conductivity ratio.
2. The number of contrarotative cells and the strength of the circulation of each cell can be controlled by the thickness of the bottom wall, the thermal conductivity ratio, and the Rayleigh number.
3. The average Nusselt number increases by increasing either the Rayleigh number and/or thermal conductivity ratio, but the average Nusselt number decreases by increasing the wall thickness.

## Figures and Tables

**Table 1:** Comparison of the average Nusselt number between the present results and other works for various Rayleigh numbers and wall-to-fluid thermal conductivity ratios.

Kr	Ra = 7.1 × 10^2^			Ra = 7.1 × 10^3^			Ra = 7.1 × 10^4^		
	1	5	10	1	5	10	1	5	10
Present	0.868	1.019	1.041	1.349	1.830	1.916	2.089	3.424	3.725
[7]	0.870	1.020	1.040	—	—	—	2.080	3.420	3.720
[18]	0.870	1.020	1.040	1.350	1.830	1.920	2.080	3.420	3.720
[19]	0.850	1.030	1.040	—	—	—	2.040	3.300	3.600

## Nomenclature

*d*, *D*: Wall thickness and dimensionless wall thickness
*g*: Gravitational acceleration
Kr: Thermal conductivity ratio
*k*: Thermal conductivity
*l*: Width and height of enclosure
$\bar{\text{Nu}}$: Average Nusselt number
Ra: Rayleigh number
*T*: Temperature
*u*, *v*: Velocity components in the *x*- and *y*-directions
*x*, *y* & *X*, *Y*: Space coordinates and dimensionless space coordinates.

### Greek Symbols

*α*: Effective thermal diffusivity
*β*: Thermal expansion coefficient
Θ: Dimensionless temperature
*ν*: Kinematic viscosity.

### Subscript

*c*: Cold
*f*: Fluid
*h*: Hot
*w*: Wall.
